# Applicability and results of the versius surgical robotic system in colorectal surgery: a systematic review of the literature

**DOI:** 10.1007/s11701-025-02336-y

**Published:** 2025-04-28

**Authors:** Stefano Gussago, Alexandre Balaphas, Emilie Liot, Guillaume Meurette, Christian Toso, Frédéric Ris, Jeremy Meyer

**Affiliations:** 1 Division of Digestive Surgery, University Hospitals of Geneva, Rue Gabrielle-Perret-Gentil 4, 1205 Geneva, Switzerland; 2 Division of General Surgery, Groupement Hospitalier de l’Ouest Lémanique, Chemin Monastier 10, 1260 Nyon, Switzerland; 3 Medical School, University of Geneva, Geneva, Switzerland

**Keywords:** Colorectal surgery, Versius Surgical Robotic System, CMR Surgical, Robotic right hemicolectomy, Robotic low anterior resection

## Abstract

Minimally invasive surgery has become the standard of care in colorectal surgery. However, the laparoscopic approach still presents technical challenges, especially when performing intracorporeal anastomosis or total mesorectal excision. Current literature underscores the potential of robotic-assisted surgery in overcoming these challenges. Among emerging robotic platforms, the Versius Surgical Robotic System, shows promises. However, its relatively recent introduction means there is a lack of data on its performances in colorectal surgery. This systematic review aimed to evaluate the current evidence on the use of Versius Surgical Robotic System in colorectal surgery. Following PRISMA 2020 guidelines, MEDLINE, CENTRAL, and EMBASE were systematically searched for original studies reporting on the use of the Versius Surgical Robotic System for colorectal surgery. Out of 199 publications screened, nine studies met the inclusion criteria for qualitative analysis. Most included studies were observational and monocentric studies, with the only large-scale, multicentric prospective data derived from the Versius surgical registry. A total of 561 colorectal procedures were reported, including oncological and benign disease, comprising 159 right hemicolectomies, 37 left colon or sigmoid resection, 253 low anterior resection and 64 abdominoperineal resections. The proportion of conversion to open surgery was 7.0% in the largest series and severe complications, defined as Clavien-Dindo grade 3 or higher was reported in 5.3% of cases. For right hemicolectomies, the mean operative time ranged from 160 to 221 min, with no conversion to open surgery reported and only one case requiring surgical revision. Intracorporeal anastomosis was reported in only one case. Regarding low anterior resection, the mean operative time ranged from 214 to 319 min, with a conversion rate to open of 6.9% and an incidence of severe complications of 6.0% in the largest series. Based on available literature there are no major safety concerns for the use of the Versius Surgical Robotic System for colorectal surgery. However, future well-conducted studies are needed to enable quantitative comparisons between different robotic platforms.

**Supplementary Information:**

The online version contains supplementary material available at 10.1007/s11701-025-02336-y.

## Introduction

Minimally invasive surgery is widely recognized as the standard of care in colorectal surgery [1, 2]. Laparoscopic surgery presents, however, several technical challenges, including a 2-dimensional view, limited range of movement, action tremors, and difficulties in camera control. These limitations are particularly relevant during low anterior resection due to the anatomic characteristics of the pelvis, but also during right colectomy due to difficulties in intra-corporeal suturing that led to a preference for anastomosis that is extracorporeal [3, 4]. In this context, interest in the robotics for colorectal surgery has been progressively increasing over the recent years. The presumed benefits of a robotic intervention over a laparoscopic one includes a stable three-dimensional view, enhanced camera control, improved dexterity thanks to reduced action tremor, and the addition of wristed instruments.

Numerous studies have explored the potential benefits of a robotic approach, notably applied to right hemicolectomy and low anterior resection. For instance, two recent systematic reviews have highlighted how robotic surgery may facilitate a fully minimally invasive right hemicolectomy, including intra-corporeal anastomosis, and offer improved postoperative recovery [5, 6]. Regarding low anterior resection, the robotic approach was reported offering better outcomes, including more primary anastomoses, a lower incidence of wound infections, improved mesorectal integrity, reduction in urinary complications, shorter hospital stays and an increased number of harvested lateral pelvic nodes [7–10].

In this context, there is growing interest in robotics applied to colorectal surgery.

The da Vinci Surgical System (Intuitive Surgicals, Sunnyvale, USA), which received approval by the Food and Drug Administration (FDA) over 20 years ago, represents the most extensively studied robotic platform. However, in recent years, new robotic platforms have emerged, including the Versius Surgical Robotic System (Cambridge Medical Robotics, Cambridge, United Kingdom), a new system that received European Conformity (CE) mark approval in 2019. Surgeons in different countries are increasingly adopting this system, but given its relatively recent development, comprehensive data on the use of this platform in colorectal surgery is limited. In this respect, the aim of this systematic review is to evaluate available literature on the application of the Versius Surgical Robotic System in colorectal surgery.

## Methods

This systematic review was conducted in accordance with the Preferred Reporting Items for Systematic Reviews and Meta-Analyses (PRISMA) guidelines [11]. MEDLINE, CENTRAL, and EMBASE were searched on 18 March, 2024, without time restrictions for original studies published in English that involved patients undergoing robotic colorectal resection using the Versius Surgical Robotic System. Additional records were identified through a manual search and reviewing the references of retrieved publications. Exclusion criteria included conference abstracts, videos, letters to the editor, secondary analyses of previously published papers, studies including patients under 18 years of age, and studies reporting on Transanal Minimally Invasive Surgery (TAMIS) or functional surgery. Details of the systematic review methodology and literature search strategy are summarized in Tables 1 and 2.

**Table 1:** Methods for the systematic review

Population	Intervention	Outcome(s)	Design
Colorectal cancer and/or benign disease	Colorectal resection using Versius Surgical Robotic System	Intra- and/or post-operative outcome(s)	Observational and/or RCT

RCT: randomized controlled trial

**Table 2:** Literature search strategy

Source of data	Search build
Database:	
MEDLINE	("Versius" AND ("robotic surgery "OR" robotic-assisted surgery"))
CENTRAL	("Versius" AND ("robotic surgery "OR" robotic-assisted surgery"))
EMBASE	("Versius" AND ("robotic surgery "OR" robotic-assisted surgery"))

Two authors (SG, JM) independently conducted a screening of the literature to identify studies that met the inclusion criteria, using the Rayyan software. Discrepancies were resolved by a third author (AB).

Data were extracted from included studies, reporting on patient demographics, type of surgery performed, operative time, console time, conversion to open or laparoscopic surgery, complications greater than Clavien-Dindo Grade 2, blood transfusions, length of hospital stay (LOS), and 30-day readmission rates.

## Results

### Inclusion process

**Fig. 1:**
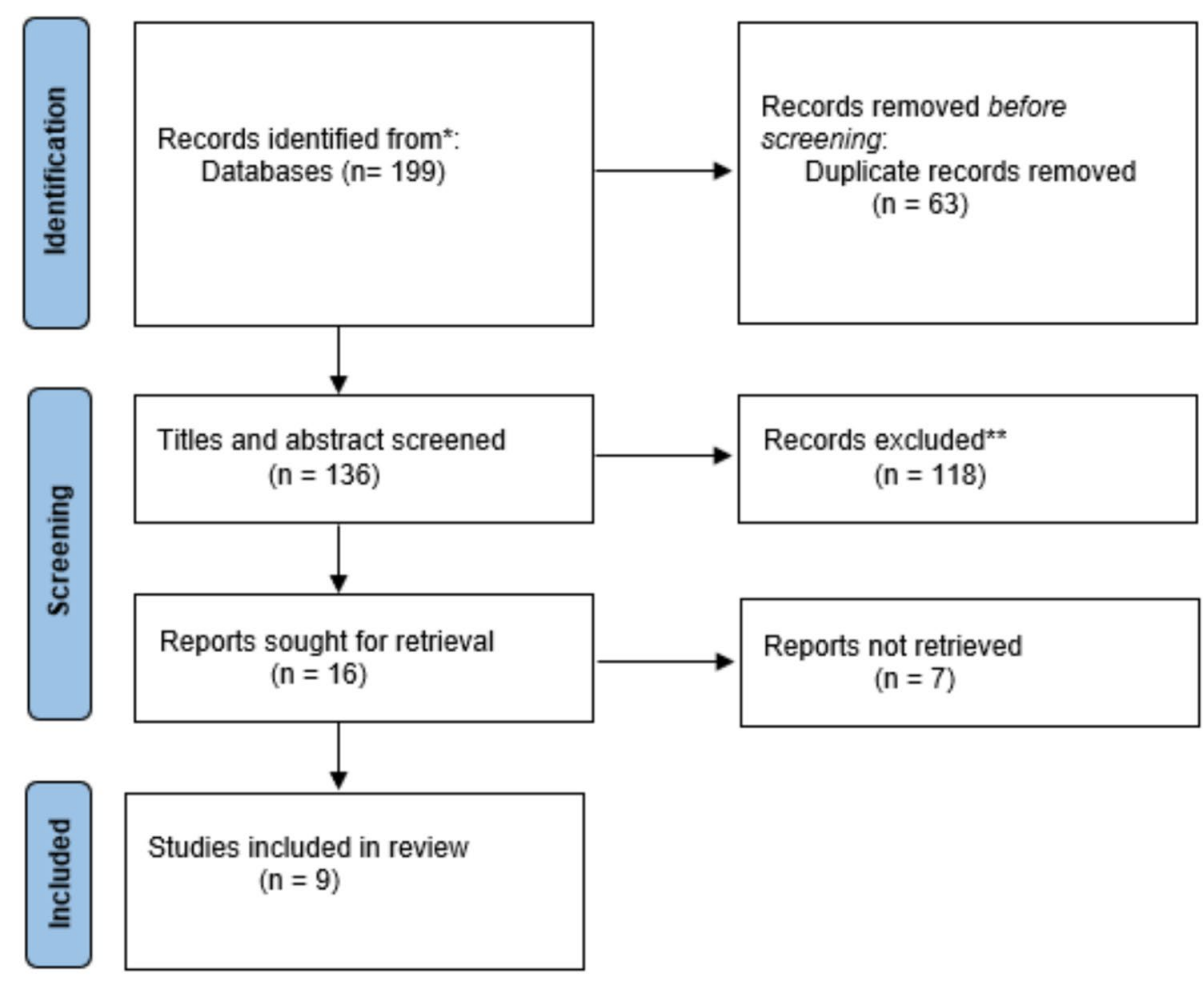
PRISMA flowchart

A total of 199 articles were identified through searches on MEDLINE, CENTRAL and EMBASE. After removing duplicates, 136 publications were screened. Based on titles and abstracts, 118 publications were excluded and 16 publications were retrieved for full-text review. Of those, seven publications were excluded and nine were included in the qualitative analysis [12–20]. The PRISMA flowchart, detailing the identification, selection, and inclusion of studies in accordance with PRISMA guidelines, is presented in Fig. 1.

### Characteristics of included studies

A summary of characteristics of includes studies is reported in Table 3. Of the nine studies included, eight were case-series [12, 14–20] and only one was a multicentric cohort study [13]. One study was based on an international registry [13]. Four studies were conducted in the United Kingdom, two in Italy, one in Germany, and one in India.

**Table 3:**
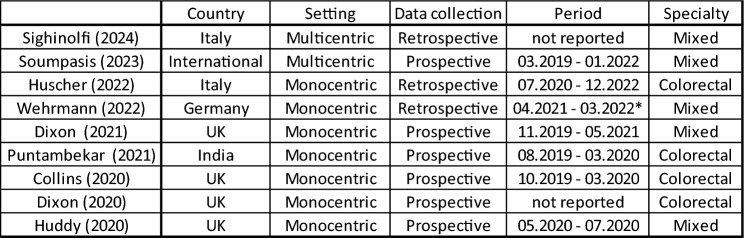
Characteristics of included studies

*Excluded period between 10.2021 and 01.2022

Four studies specifically focused on colorectal surgery [14, 17–19], while the remaining studies reported a broader range of procedures, including urological and gynaecological surgeries.

The number of patients included across studies varied widely, ranging from 1 to 374 for colorectal procedures alone [12, 13].

### Population and surgical procedures

Seven studies reported the mean age of patients, which ranged from 55.6 to 68 years [13–19], and six studies documented the average BMI, which ranged from 23.6 to 28.9 kg/m^2^ [13–17, 19]. Six studies also reported the gender distribution, with a predominance of male patients [13, 14, 16–19]. The indication for surgery—whether oncological vs non-oncological—was documented in six studies, with oncological cases being the primary indication in most instances [14, 16–20]. The American Society of Anaesthesiologists (ASA) classification was reported in five studies for ASA II status [13, 14, 16, 17, 19] and in six studies for ASA III status [13, 14, 16–19]. In the largest series, ASA II patients accounted for 67.2% (248/369) of cases, while ASA II patients accounted for 9.2% (34/369). Only three studies reported a history of previous surgery in the patient population [13, 14, 16] (Table 4).

**Table 4:**
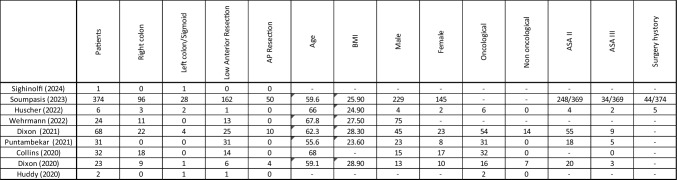
Procedures and patients carachteristics

AP: abdominoperineal, BMI: body mass index, ASA: American Society of Anaesthesiologists

Of the total of 561 cases, right hemicolectomy and low anterior resection of the rectum were the most representatives, accounting for 159 and 253 cases, respectively. Other procedures, such as sigmoidectomy or left hemicolectomy and abdominoperineal resection, were less common, accounting for 37 and 64 cases, respectively.

### Intraoperative and postoperative outcomes

Four studies reported the mean operative time, which ranged from 197.5 to 294.7 min [13–15, 18]. The console time was documented in four studies, ranging from 51 to 166 min [12, 16–19] (Table 5). In the largest series by Soumpasis, which included 374 procedures, 26 (7.0%) cases required conversion to an open approach, and 24 (6.5%) to laparoscopic surgery. These findings were consistent with the studies by Collins and Dixon (2021), who reported conversion rates to open surgery of 4.4% and 6.25%, respectively [16, 18]. Regarding postoperative outcomes, all included studies reported the incidence of severe complications, defined as Clavien-Dindo Grade 3 or higher. A total of 26 severe complications were reported, 16 of which required surgical revision [12–20]. Only three studies reported the need for blood transfusion [12–14]. The length of stay (LOS) was reported in eight studies, with mean (or median) LOS ranging from 2 to 9.2 days [12–19]. Readmission within 30 days was reported in five studies, with Soumpasis documenting 11 (4.1%) cases out of 267 patients [13, 14]. LOS: Length of stay, BSU Bed Side Unit

**Table 5:**
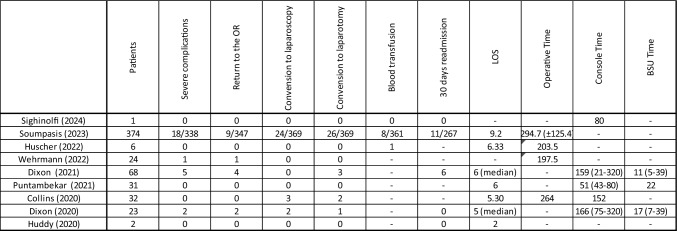
Outcomes of colorectal surgery (all mixed)

LOS: Length of stay, BSU Bed Side Unit

### Right hemicolectomy

Specific outcomes for right hemicolectomy were reported by five studies [13–16, 18, 19] (Table 6). Three studies described the technique of anastomosis in 32 patients, with intra-corporeal anastomosis performed in only one patient (3.13%) [14, 15, 18]. The number of harvested lymph nodes was reported in one study, with an average of 14.3 lymph nodes [14]. Three studies reported the mean operative time for right hemicolectomy, which ranged from 160 to 221 min [14, 15, 18]. Console time was documented in two studies, with mean console times ranging between 111 and 154 min [18, 19]. No conversions to open surgery were reported in the included studies, although two cases required conversion to a laparoscopic procedure [18]. One severe complication, requiring surgical revision, was reported [15].

**Table 6:**
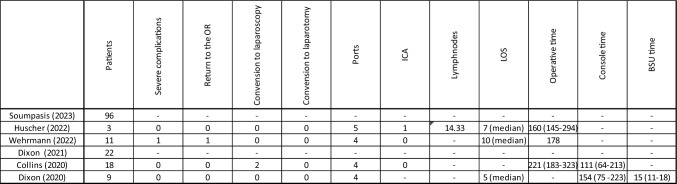
Outcomes of right hemicolectomy subgroup

ICA: intra-corporeal anastomosis, LOS: Length of stay, BSU Bed Side Unit

### Low anterior resection of rectum

Specific outcomes of low anterior resection of the rectum were reported by eight studies, with the largest series comprising 162 cases [13–20]. Four studies reported the mean operative time, which ranged from 214 to 319 min, [13–15, 18]. Two studies documented console time, with mean durations of 51 and 204 min [17, 18].

Three studies addressed the quality of the specimen, with 27 (87.1%) cases of complete mesorectal excision and 4 (12.9%) cases of nearly complete mesorectal excision among 31 patients in the largest series [14, 15, 17].

Six studies reported conversion to open surgery, ranging from 0% to 14.3%, while conversion to laparoscopy was documented at 5.0% and 7.1% in the series of Sumpasis and Collins, respectively. [13–15, 17, 18, 20].

A total of nine severe complications were described, with four patients requiring a surgical revision. The length of stay was described by five studies and ranged from 2 days to a mean 9.2 days [13–15, 17, 20]. Outcomes of low anterior resection of the rectum are reported in Table 7.

**Table 7:**
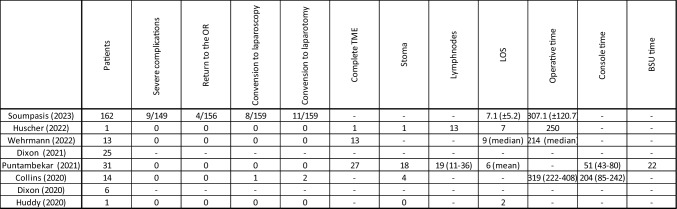
Outcomes of anterior resection of the rectum subgroup

LOS Length of stay, BSU Bed Side Unit, TME Total Mesorectal Excision

## Discussion

The Versius platform has been recently introduced into the market. As highlighted by our systematic review, the majority of available studies are heterogeneous in terms of patient populations, interventions and reported outcomes. Noteworthy, most of them report outcomes of surgical procedures from different surgical specialties. Additionally, a certain percentage of patients might potentially be duplicates, given that one of the included studies is a multicentre international registry.

Regarding population characteristics, most of the patients included are middle-aged, overweight, males, in relatively good health, with oncological indications for surgery. These characteristics are consistent with other studies comparing laparoscopic versus robotic approach in colorectal surgery [7, 8].

In our review, we decided to include all cases of colic resection, regardless of whether the indication was benign or oncological in nature. Regarding the latter, only Puntambekar described the pre-operative oncological characteristics in his work, with significant variability in tumor location in the inclusion criteria, with the primary localisation ranging from 3 to 18 cm from the anal verge [17].

Unsurprisingly, right hemicolectomy accounted for 159 cases and anterior rectal resection for 253 cases. During robotic right hemicolectomy using the Versius system, no conversion to open surgery was reported. However, conversion to laparoscopy was necessary in two cases. As reported by Collins, the conversion was required in one case for bleeding control due to a slipped off Hem-o-lock, and in a second case to assess abnormal anatomy where the sigmoid colon was retroperitoneal and lay close to duodenum. [18].

The mean operating time varied from 160 to 221 min, consistent with other series reporting on robotic right hemicolectomy [5].

A significant challenge in comparing the Versius Surgical Robotic System to other robotic platforms is that the definition of laparoscopic conversion is not clearly defined. In fact, this system currently lacks integrated robotic stapling and advanced bipolar devices, which means that the procedure is, by definition, at least laparoscopically-assisted. This is intrinsic to the design of the Versius Surgical Robotic System, which is intended to support laparoscopic surgery rather than replace it. Contrary to other systems, the Versius system requires no specific robotic ports, allowing for alignment during docking and standard laparoscopic placement.

In the three studies reporting on suturing techniques, intracorporeal anastomosis was performed in only one patient [14, 15, 18]. This unfortunately results in the need for a midline incision, which increases the patients risk of postoperative pain, surgical site infection, post-operative ileus, and incisional hernia [21–25]. This is somehow surprising given that one of the claimed advantages of robotic surgery is the improved dexterity in suturing and the possibility to easily perform intracorporal anastomosis, and may reflect the relative unfamiliarity of surgeons with the platform, as these studies are all"case series"with a limited number of patients and represent initial implementation of the Versius system.

Another advantage of robotic surgery is that it may facilitate central lymph node dissection enabling an increased number of lymph nodes to be harvested. However, only one case series, which included three patients, reported an average of 14.33 lymph nodes harvested – an insufficient sample size to draw firm conclusions [14].

Eight studies provided specific data on low anterior resection. In the largest series by Soumpasis et al., which included 162 patients, conversion to laparotomy or laparoscopy were required in 6.9% and 5.0% of the procedures, respectively. These conversion rates are slightly higher than those reported in other series [7, 8]. This could reflect the limited familiarity of the operator with the Versius platform and the absence of articulated vessel sealer and stapler.

Moreover, in three of the four studies that detailed the operating technique, splenic flexure mobilization (SFM) was frequently performed laparoscopically [14, 15, 17, 18]. In particular, Puntambekar described a systematic laparoscopic mobilization of the colic flexure in his technique. As noted by various authors, performing SFM in robotic surgery can be challenging, as it may prolong operating time and potentially require redocking, especially in the absence of motion-activated operating tables [26, 27]. In this context, both Collins and Wehrmann found a certain limitation of the Versius Surgical Robotic System in the opportunity of changing space during the procedure, emphasizing the importance of a careful planning of port placement [15, 18].

Only one study reported that nine patients (6.0%) experienced severe complications (Clavien-Dindo > 2), in line with the results reported by Shadmanov, but significantly lower than the 18.4% reported by Burghgraef et al. [7, 8, 13]. In the same series, four patients (2.6%) required surgical reintervention, also lower than the 16.5% reported by Burghgraef, but in line with the 2.6% found in the work of Khan et al. [6, 28]. Histopathological outcomes, including the quality of total mesorectal excision (TME), were reported in three studies, with a complete mesorectal excision in 87.1% of cases (27 of 31) reported by Puntambekar. While clearly superior compared to the 71.7% reported by Burghgraef et al., in this single study, the Versius system failed to reach the percentages achieved in the work of Shadmanov et al. and de'Angelis of 98.9% and 97.3%, respectively [7, 8, 29]. However, it is interesting to note that in the work of the European MRI and Rectal Cancer Surgery (EuMaRCS) Study Group, the percentage of complete laparoscopic mesorectal excision stood at 78.3% [29]. These findings suggest that rectal resection using the Versius Surgical Robotic System seems safe from both intraoperative and histopathological perspectives.

This systematic review has several limitations. Most of the included articles are case-series, many of them retrospectives, which introduces a significant risk of bias in terms of patient selection. As reported by Wehrmann, patient inclusion was progressively “opened” to more complex cases during the study, starting with robotic cholecystectomy before moving on to right-sided colectomy and anterior resection of the rectum [15]. This reflects the relatively recent introduction of the Versius Surgical Robotic System to the market and the fact that some of the studies are implementation studies or preliminary experiences with this robotic system [14–16, 18, 19]. These factors likely contribute to the longer operating time and a greater tendency to convert to laparoscopy and laparotomy. However, it should be noted that due to the characteristics of the platform itself, conversion to laparoscopy does not require port transposition, allowing the procedure to continue without impact on the patient.

Secondarily, many of the studies included in our systematic review had gaps in reporting preoperative patient characteristics. In particular, the largest series by Soumpasis, did not even mention whether the operative indication was benign or malignant [13]. Even when preoperative oncological characteristics were reported, as in the case of Puntambekar's series, they exhibited wide variability, making the population under review highly heterogeneous [17]. This naturally reduces the impact of the oncological analysis compared to studies with a population that has more uniform preoperative characteristics.

Despite these limitations, the current data are encouraging regarding the application of the Versius Surgical Robotic System in colorectal surgery, particularly in terms of intraoperative safety and operative specimen quality outcomes.

Further studies, especially those involving a wider deployment of the platform and/or its instruments, are essential to better understand the impact of the Versius Surgical Robotic System in colorectal surgery.

## Supplementary Information

Below is the link to the electronic supplementary material.

Supplementary file1 (XLSX 10 KB)

Supplementary file2 (XLSX 10 KB)

Supplementary file3 (XLSX 9 KB)

Supplementary file4 (XLSX 10 KB)

Supplementary file5 (XLSX 10 KB)

Supplementary file6 (XLSX 9 KB)

Supplementary file7 (XLSX 10 KB)
